# The Impact of Nozzle Opening Thickness on Flow Characteristics and Primary Electron Beam Scattering in an Environmental Scanning Electron Microscope

**DOI:** 10.3390/s24072166

**Published:** 2024-03-28

**Authors:** Jiří Maxa, Pavla Šabacká, Jan Mazal, Vilém Neděla, Tomáš Binar, Petr Bača, Jaroslav Talár, Robert Bayer, Pavel Čudek

**Affiliations:** 1 Institute of Scientific Instruments of the CAS, Královopolská 147, 612 64 Brno, Czech Republic; 2 Faculty of Electrical Engineering and Communication, Brno University of Technology, Technická 10, 616 00 Brno, Czech Republicrobert.bayer@vut.cz (R.B.);; 3 Faculty of Military Robotics, University of Defence, 662 10 Brno, Czech Republic; 4 Faculty of Military Leadership, University of Defence, 662 10 Brno, Czech Republicjaroslav.talar@unob.cz (J.T.)

**Keywords:** Ansys Fluent, ESEM, critical flow, nozzle, CFD, numerical simulation

## Abstract

This paper describes the methodology of combining experimental measurements with mathematical–physics analyses in the investigation of flow in the aperture and nozzle. The aperture and nozzle separate the differentially pumped chamber from the specimen chamber in an environmental scanning electron microscope (ESEM). Experimental measurements are provided by temperature and pressure sensors that meet the demanding conditions of cryogenic temperature zones and low pressures. This aperture maintains the required pressure difference between the chambers. Since it separates the large pressure gradient, critical flow occurs on it and supersonic gas flow with the characteristic properties of critical flow in the state variables occurs behind it. As a primary electron beam passes through the differential pumped chamber and the given aperture, the aperture is equipped with a nozzle. The shape of the nozzle strongly influences the character of the supersonic flow. The course of state variables is also strongly influenced by this shape; thus, it affects the number of collisions the primary beam’s electrons have with gas molecules, and so the resulting image. This paper describes experimental measurements made using sensors under laboratory conditions in a specially created experimental chamber. Then, validation using mathematical–physical analysis in the Ansys Fluent system is described.

## 1. Introduction

This paper deals with the issue of pumping environmental scanning electron microscope (ESEM) vacuum chambers. This paper also deals with the issue of setting and monitoring (sensing) chamber pressures. Proper adjustment of the shapes of the nozzle section and chambers will guarantee the desired pressure distribution. The pressure distribution can be verified by sensors at defined locations in the chamber. As the problem is nonlinear, it is necessary to include numerical modeling in the area of pressure sensing and distribution. This is a hybrid measurement method that combines pressure sensing and a precise numerical model [1,2,3]. This paper deals with experimental measurements of an electron microscope under laboratory conditions (ISI CAS, Brno, Czech Republic). A conventional electron microscope must work in a vacuum to eliminate electron beam scattering. For this reason, it does not allow for the observation of wet samples that would immediately dry and be destroyed in a vacuum. Therefore, a special kind of electron microscope was developed (ESEM). The key element of an ESEM is a higher pressure level in the specimen chamber to preserve wet samples. For this reason, the specimen chamber is separated from the tube by a differentially pumped chamber. These chambers are separated by a very small aperture or nozzle, which allows for separating the chambers at very different pressures. The basic principle of an ESEM is described in more detail in the following section.

The main contribution of this paper is research on the impact of the shape of this aperture/nozzle on the functionality of the microscope. Since the shape strongly influences the pressure distribution in the differentially pumped chamber through which the primary electron beam passes, it has a major impact on the resulting image sharpness. The influence of this aperture/nozzle is very significant. As it separates two spaces with a large pressure gradient, critical flow, supersonic flow, a Mach disk, and shock waves occur on it. All this causes large pressure gradients that significantly affect conditions influencing the passage of the electron beam. This is because the beam passes through the differentially pumped chamber, and further through the aperture into the specimen chamber to the sample itself.

Dr. Danilatos has been researching this issue for a long time [4]. In the article “Beam transfer characteristics of a commercial environmental SEM and a low vacuum SEM” [5], it is possible to become acquainted with the basic study of density and pressure courses in gas flow through a small aperture separating two chambers with a large pressure gradient. In this paper, Dr. Danilatos uses the example of an FEI electron microscope (now ThermoFisher) to analyze the gas flow through the aperture depending on its size and controlled back pressure. Furthermore, Dr. Danilatos, in his publication “Figure of Merit for Environmental SEM and its Implications” [6], deals with the size of the angle and thickness of the nozzle without aperture on the given problematics. He concludes with the advantage of a larger nozzle opening and the contradictory conditions in terms of its thickness. The thinnest possible nozzle promotes optimal passage of the primary electron beam. On the other hand, for the vacuum pumps to achieve a sufficient pressure gradient between the chambers separated by the aperture, the thickest possible nozzle is needed.

In another publication, “Optimum beam transfer in the environmental scanning electron microscope” [7], he also deals with the optimization of electron beam passage depending on the density distribution on its path. He points out that the thin nozzle represents the minimum thickness of increased pressure that the electron beam must overcome during its transmission.

The given analyses are followed by studying the nature of the flow and its effect on the passage of the primary electron beam. These analyses were also performed for the chambers in the microscope separated by a small aperture with an attached nozzle. It combines the previously mentioned contradictory requirements for this construction [8]. The aperture separates the chambers by critical flow for better pumping. The desired pressure gradient and the nozzle allow for controlling the expansion of the gas above the aperture. So, it suitably affects the pressure and density distribution of the gas through which the primary electron beam passes [9,10]. The so-called calculation cross-section of the nozzle, at a common angle of 12° of the de Laval nozzle, was designed. The calculation cross-section of the nozzle was compared with other opening angles, either for over-expanded or under-expanded nozzles. The following nozzle opening variants were selected for the analysis: 8°, 10°, 12°, 14°, 16°, and 18°. The results were evaluated concerning the basic analysis of electron scattering.

These analyses were used as a basis for the upcoming experiment on an experimental chamber simulating the work of a given part of the microscope, which will be advantageous for the subsequent differentially pumped chamber design of the next generation of ESEM.

In this paper, an analysis of the impact of the nozzle opening behind the aperture on the character of the flow in the nozzle is performed. The result is a different course of the pressure quantity. The value of static pressure has a significant effect on the passing electron beam’s scattering. Beam dispersion is one of the key values influencing the resulting sharpness of the image in an ESEM. The beam dispersion for individual variants is evaluated based on the obtained results.

## 2. Environmental Scanning Electron Microscope (ESEM)

**Figure 1:**
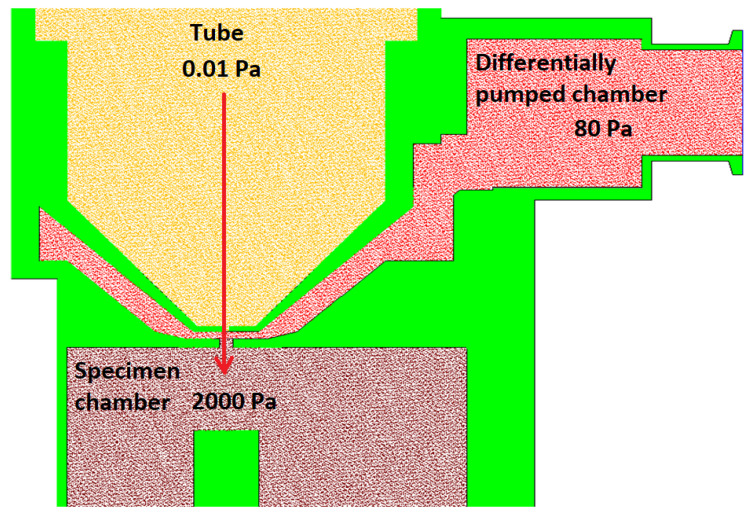
Environmental scanning electron microscope (ESEM)—chamber diagram.

Electron microscopy has brought the possibility of monitoring without the necessity of a light source and zooms at several times more detail than conventional optical microscopes. The environmental scanning electron microscope (ESEM) has been developed, which does not impose such requirements on the vacuum. With an ESEM, it is possible to observe electrically non-conductive, semiconducting samples [11] or native samples [12,13] without damaging them or to study these samples in dynamic experiments in situ [14,15,16]. Signal electrons are detected in an ESEM by particular ionization or scintillation detectors [17,18]. ESEMs differ from the conventional type of electron microscope by adding a differentially pumped chamber. This chamber separates the specimen chamber from the tube. Due to the large pressure difference in the tube (0.01 Pa) and specimen chamber (usually around 600–2000 Pa), these two chambers cannot be separated by only one aperture. Otherwise, it would not be possible to achieve such a low pressure in the tube by pumping [19]. Therefore, in an ESEM, a differentially pumped chamber is inserted into this interspace, which can separate these pressure differences by separating the tube and the specimen chamber. Each chamber is separated by a small aperture. The pressure drop created between the differentially pumped chamber and the tube is approximately from 80 Pa to 0.01 Pa. Subsequently, another pressure drop is created by the specimen chamber and the differentially pumped chamber at a ratio of 2000 Pa to 80 Pa. This state is shown schematically in Figure 1. The values in the figure are only indicative. The exact boundary conditions for the described analyses are given in below.

**Figure 2:**
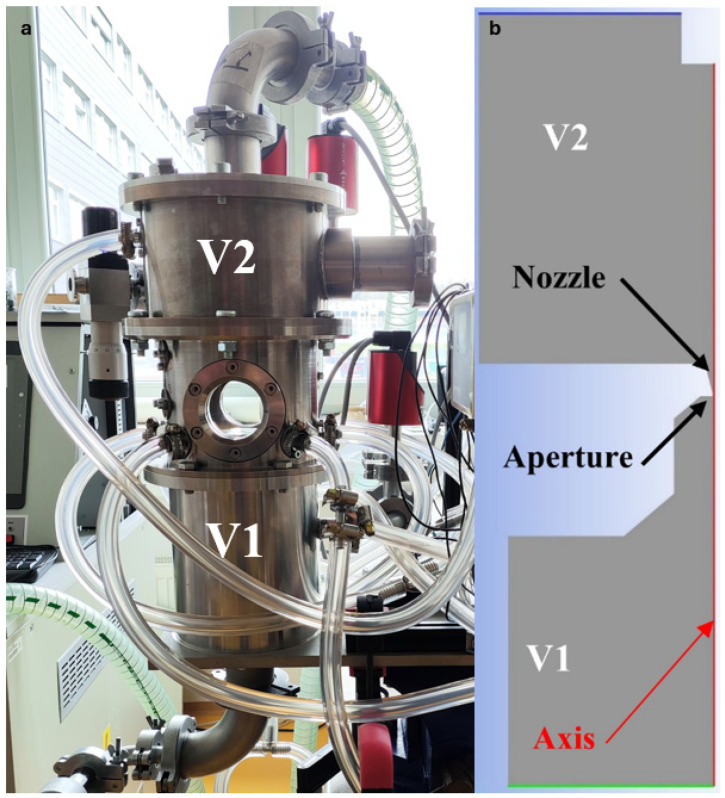
Real experimental chamber (**a**) compared with simplified 2D axisymmetric model of the experimental chamber (**b**).

Recently, an experimental chamber simulating pumping conditions in an electron microscope environment was constructed (Figure 2—real experimental chamber (Figure 2a) compared with a simplified 2D axisymmetric model of the chamber focused on flow, mainly in aperture and nozzle flow (Figure 2b)). This experimental chamber was developed by the team of Vilém Neděla in ISI CAS. It was lent to Brno University of Technology. In this experimental chamber, two chambers with a large pressure gradient are separated by a small differentially pumped region in the shape of a nozzle [20,21,22].

## 3. Theory of One-Dimensional Isentropic Flow

As mentioned, the ESEM is characterized by its construction. Chambers with large pressure gradients are separated by a small aperture. This causes the so-called critical flow. If we have areas with different pressures, the flow runs from the chamber of higher pressure to the chamber with lower pressure. So, there is a flow of a certain velocity in the aperture. In the aperture, the velocity of the flow is proportional to the increasing pressure difference on both sides of the aperture [23]. This ratio is valid only until the aperture flow velocity reaches 1 Mach. At the given moment, so-called critical flow occurs when the speed of the flow in the aperture cannot take values greater than 1 Mach. Exceeding this value will not be achieved even by further increasing the pressure difference on both sides of the aperture. Similarly, no greater mass flow rate per unit of time can pass through the aperture than the amount that passes through the aperture at the moment when the gas in the aperture flows at 1 Mach.

Behind the aperture, supersonic flow is formed, in which there is a certain area of reduced pressure. This area beyond the aperture arises because only the amount of gas that passes through the aperture at a speed adequate to the speed of sound, as already described, can pass through the aperture. The supersonic flow region is terminated by some type of shock wave. This is an area with increased gas density [24].

In the described type of aperture flow, there are dependencies of state variables such as pressure, temperature, density, velocity, and Mach number, which describe the theory of one-dimensional isentropic flow.

These state variables and their mutual relations are described using the following equations (Equations (1)–(6)) [25]:

(1) $$\frac{v_{v}}{v_{kr}}={\left[\frac{\left(\varkappa +1\right)M^{2}}{2+\left(\varkappa -1\right)M^{2}}\right]}^{\frac{1}{2}}$$

(2) $$\frac{v_{v}}{v_{0}}={\left[\frac{2}{2+\left(\varkappa -1\right)M^{2}}\right]}^{\frac{1}{2}}$$

(3) $$\frac{T_{v}}{T_{0}}=\frac{2}{2+\left(\varkappa -1\right)M^{2}}$$

(4) $$\frac{p_{v}}{p_{0}}={\left[\frac{2}{2+\left(\varkappa -1\right)M^{2}}\right]}^{\frac{\varkappa }{\varkappa -1}}$$

(5) $$\frac{\rho_{v}}{\rho_{0}}={\left[\frac{2}{2+\left(\varkappa -1\right)M^{2}}\right]}^{\frac{1}{\varkappa -1}}$$

(6) $$\frac{\rho_{v}}{\rho_{kr}}=\frac{A_{kr}}{A}=M{\left[\frac{\varkappa +1}{2+\left(\varkappa -1\right)M^{2}}\right]}^{\frac{1}{2}\frac{\varkappa +1}{\varkappa -1}}$$

where *p*_0_ is the input pressure, *p_v_* is the output pressure, *T*_0_ is the input temperature, *T_v_* is the output temperature, *v*_0_ is the input velocity, *v_v_* is the output velocity, *v_kr_* is the critical velocity, *ρ*_0_ is the input density, *ρ_v_* is the output density, *M* is the Mach number, ϰ is the gas constant = 1.4, *A* is the computational cross-section, and *A_kr_* is the critical cross-section.

## 4. Methodology

Using the theory of one-dimensional isentropic flow, the computational cross-section of the nozzle for the angle of 12° was determined. The angle determination, described in Section 5.1, ensures controlled gas expansion behind the nozzle [26,27,28,29,30].

Based on the previous steps, the flow pattern of this nozzle was analyzed. The pattern analysis was made in the third step on a tuned mathematical–physical model with the calculation cross-section of the nozzle at a common angle of 12° of the Laval nozzle [31]. This calculation state of the nozzle was then compared with other opening angles, either for over-expanded or under-expanded nozzles. So, this made for a series of opening angles: 8°, 10°, 12°, 14°, 16°, and 18°.

Finally, results concerning the basic analysis of electron scattering were evaluated. The opening angle has a significant impact on the function of the microscope, and the results are one of the bases for the construction of differentially pumped chambers.

The Ansys Fluent system (ANSYS, Inc., San Jose, CA, USA) uses Stokes–Navier equations for its calculations. These are partial nonlinear differential equations of the second order. These equations cover all aspects of real-world fluid behavior, including turbulence. The equations were solved by the finite volume method.

Stokes–Navier equation in component formulation:

(7) $$\frac{Du_{i}}{D_{t}}=\frac{\partial u_{i}}{\partial t}+u_{k}\frac{\partial u_{i}}{\partial x_{k}}=-\frac{1}{\varrho }\frac{\partial p}{\partial x_{i}}+\upsilon \frac{\partial^{2}u_{i}}{\partial x_{k}\partial x_{k}}$$

The physical importance of individual terms of a Stokes–Navier equation is defined as follows:

$\frac{\partial u_{i}}{\partial t}$ variability of the flow field in time;

$u_{k}\frac{\partial u_{i}}{\partial x_{k}}$ characterizes convection;

$-\frac{1}{\varrho }\frac{\partial p}{\partial x_{i}}$ pressure gradient;

$\upsilon \frac{\partial^{2}u_{i}}{\partial x_{k}\partial x_{k}}$ effect of viscosity.

Transport energy equation:

(8) $$\frac{\partial \left(\varrho E\right)}{\varrho t}+\nabla \left[V\left(\varrho E+p\right)\right]=\nabla \left[k_{eff}\nabla T-\underset{j}{\sum }h_{j}J_{j}+\tau_{eff}V\right]+S_{h}$$

where *k_eff_* is the effective conductivity of the fluid and ***J****_j_* is the diffusion flux of element *j*. The first three terms on the right-hand side of the equation represent energy transfer due to conduction, element diffusion, and viscous dissipation.

$\frac{\partial \left(\varrho E\right)}{\varrho t}$ change in density and energy over time;

$\nabla \left[V\left(\varrho E+p\right)\right]$ heat transfer through the line (inlet and outlet);

$k_{eff}\nabla T$ heat transfer through the conduction inside the system;

$\sum_{j}h_{j}J_{j}$ heat transfer by diffusion;

$\tau_{eff}V$ viscous dissipation;

$S_{h}$ enthalpy of the system.

Energy *E* per mass unit:

(9) $$E=h-\frac{p}{\varrho }+\frac{V^{2}}{2}$$

where *E* is energy, *T* is time, *ρ* is density, ***V*** is velocity, and *p* is pressure [32].

Experience and results obtained using the theory of one-dimensional isentropic flow assume the occurrence of large pressure gradients, which also induce large temperature gradients [33]. Therefore, the density-based solver setting has proven itself for this type of analysis. It is a solver based on the density of the control equation of continuity, momentum, energy, and transport of substances simultaneously as a set of equations. Control equations for other scalars are solved sequentially, i.e., separated from each other and from the connected set for solving the conjugate set of equations. Due to the complexity of the flow in the nozzle, the solver showed a more economical choice of the implicit formulation of linearization of conjugate equations. Each equation in the conjugate set of control equations is linearized implicitly with respect to all dependent variables in the set. The pooled implicit approach resolves all variables in all cells simultaneously. This choice proved stable and suitable for the complex case of large pressure gradients in supersonic flow with a significant pressure drop when pumping the experimental microscope chamber.

In the next setting, the advection upstream splitting method (AUSM) scheme was selected. In this improved method, convective and compressive flows are formulated using the eigenvalues of the Jacobian flow matrices.

The AUSM has many features. The main features are as follows:

- Accurate capturing of shock and contact discontinuities.
- Entropy-satisfying solution.
- Free of “carbuncle” phenomena—the carbuncle phenomenon is a shock instability that appears when numerical low-dissipative shock-capturing techniques are used.
- Uniform accuracy and convergence rate for all Mach numbers.

Since the method does not specifically require eigenvectors, it is especially attractive for the system whose eigenstructure is not known explicitly, as in the case of two-fluid equations for multiphase flow.

To solve the transfer of results between cell meshes, the second-order upwind scheme was chosen. The variables on cell surfaces are calculated using a multivariate linear reconstruction approach [34]. In this approach, higher-order accuracy is achieved at the cell faces using the Taylor series of expansion of a cell-centered solution around the cell’s center of gravity [35,36,37].

The given setting was suitable for dealing with the flow with all the changes induced during pumping and fully managed this type of very complex flow. So, it corresponded to the results of the experimental measurements. The given mathematical–physics analysis required a suitably chosen mesh.

**Figure 3:**
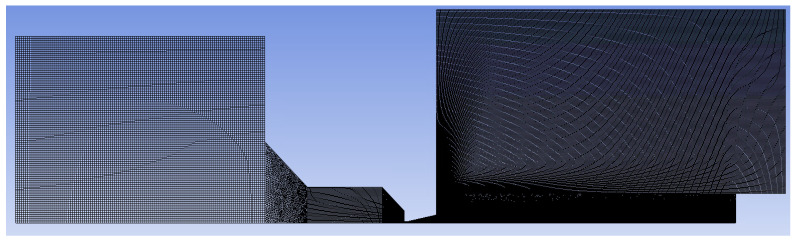
Structured mesh for mathematical–physics analysis.

**Figure 4:**
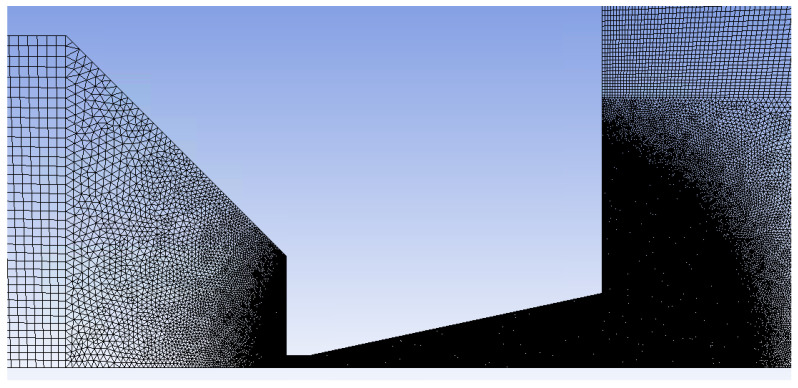
Zoomed area with mesh refinement.

A combination of structured mesh with a 2D variant of hexagonal elements was made. This method is known for its advantage of reduced blurred results caused by a possible error when transmitting results over oblique edges. At the same time, this method saves the number of cells when meshing purely rectangular areas (Figure 3). In areas where it was not possible to use quality structured mesh, triangles were used. These are narrowed areas, especially in the aperture itself, the nozzle, and behind the nozzle with the expected supersonic flow. Here, a large refinement of the grid was made (Figure 4) due to the expected large gradients and shock waves [38].

In addition, during the calculation, manual adaptive refinement was performed using the field variable method. The choice of mesh adaptation range was selected according to the maximum values in the cell-derivative option gradient pressure with maximum refinement level 4. As a result, pressure gradients in the supersonic flow areas in the nozzle were appropriately captured.

The low-pressure boundary slip setting has already been successfully tested and tuned in previous analyses [39]—slip flow mode is set on the walls in Ansys Fluent, respecting the lower pressure condition using Maxwell’s model [40]:

(10) $$U_{w}-U_{g}=\left(\frac{2-\alpha_{v}}{\alpha_{v}}\right)K_{n}L_{c}\frac{\partial U}{\partial n}\approx \left(\frac{2-\alpha_{v}}{\alpha_{v}}\right)\frac{\lambda }{\delta }\left(U_{g}-U_{c}\right)$$

(11) $$V_{g}≣{\left(\vec{V}\vec{n}\right)}_{g}=V_{w}$$

where *U* and *V* are defined as components of velocity that are parallel and perpendicular to the wall. The indices *g, w*, and *c* indicate the gas velocities, the walls, and the center of the cell. *δ* is the distance from the center of the cell to the wall. *L_c_* is the characteristic length. *α_v_* is the adjustment coefficient of the gas mixture and its value is calculated as the mass-weighted average of each gas in the system.

This option was chosen as an evaluation of the solution of the given mathematical Maxwell model for ESEM conditions with the expected low-slip effect.

Last, a grid independence study was performed. Manual adaptation of the mesh for the cell in range version for the entire pressure range was used again for areas with minimal variable changes with the choice of maximum refinement level 2 and area before entering the aperture, in the nozzle, and the area of gas expansion with the choice of maximum refinement level 4.

During the next course of the calculation, there was no further change in monitoring in the Ansys Fluent system of the set Monitors. Global parameters—absolute pressure, static temperature, velocity, density, and parameter points—were monitored at selected points in the aperture throat, and five points of 2 mm each in the direction of the gas flow above the aperture.

Similarly, the subsequently evaluated courses of quantities overlapped, as is presented further in this paper.

The analysis of grid independence showed compliance, making it a sufficiently fine mesh for the type of analysis.

## 5. Results and Discussion

**Figure 5:**
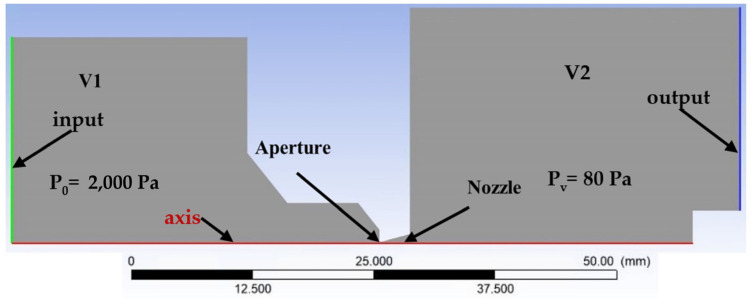
Two-dimensional axisymmetric model of chambers for mathematical–physics analysis with labeled boundary and initial conditions.

Figure 5 shows the chamber for 2D axisymmetric mathematical–physics analysis with marked boundary conditions. The volume of chamber V1, which simulates the specimen chamber, is 303,849.56 mm^3^. The volume of chamber V2, which simulates a differentially pumped chamber, is 456,186.51 mm^3^. The axis of the symmetry is indicated by red. The green line is the input with boundary conditions of 2000 Pa. The blue line is the output with boundary conditions of 80 Pa.

### 5.1. Calculating the Cross-Section of the Nozzle

In the first step, a computational cross-section of the simplified Laval nozzle shape was designed, which ensures that the shape of the nozzle exactly follows the shape of the supersonic flow beyond the critical cross-section, so that there is no additional expansion or compression at the end of the nozzle.

**Figure 6:**
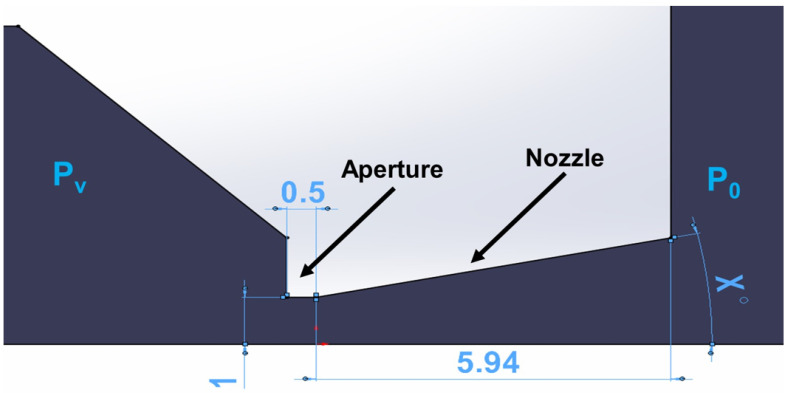
Two-dimensional axisymmetric shape of the nozzle for calculation with its dimensions [mm].

According to [39], an opening angle of 12° was chosen. From the relationships of isentropic one-dimensional flow (Equations (1)–(6)), the Mach number at the output *M_v_* was first determined from the ratios of pressure gradient *P*_0_ and *P_v_*, where *P*_0_ is 2000 Pa and *P_v_* is 80 Pa as back pressure (Figure 6). Furthermore, from Equation (12), the speed of sound in the given environment is determined and further, from Equation (2), we obtain the theoretical speed at the output *v_v_* as the product of *M_v_* and *A_v_*:

(12) $$v_{0}=\sqrt{\chi RT_{0}}$$

where *R* is the universal gas constant and *T*_0_ = 297.15 K.

The other quantities in Table 1 were obtained from Equations (1)–(6).

The output cross-section *A_v_* is obtained from Equation (6) when the critical cross-section with an aperture diameter of 2 mm is 3.14 mm^2^.

The size of the *A_v_* is 10.488 mm^2^, so the diameter of the output throat is 3.65 mm. This cross-section was corrected to a diameter of 4.4 mm concerning the actual flow profile when the velocity of the flow at the wall under normal no-slip conditions decreases to zero. In our case, there is a slip flow in low pressure, which was solved in [39].

The dimensions of the designed nozzle shape are shown in Figure 6. The nozzle is shown as a 2D axisymmetric shape for calculation in Figure 6. The angle X is 12° in this case.

For control, the Ansys Fluent system analyzed mass flow in the critical aperture cross-section and at the nozzle output throat. Both come out identically 1204 × 10^−5^ kg·s^−1^.

Table 1 shows the results obtained from Equations (1)–(6), which lead to the determination of the output cross-section of the nozzle. In this way, the correctness of the mathematical–physics analyses calculations was verified using the theory of isentropic one-dimensional flow. These calculations were the basis for experimental measurements.

**Figure 7:**
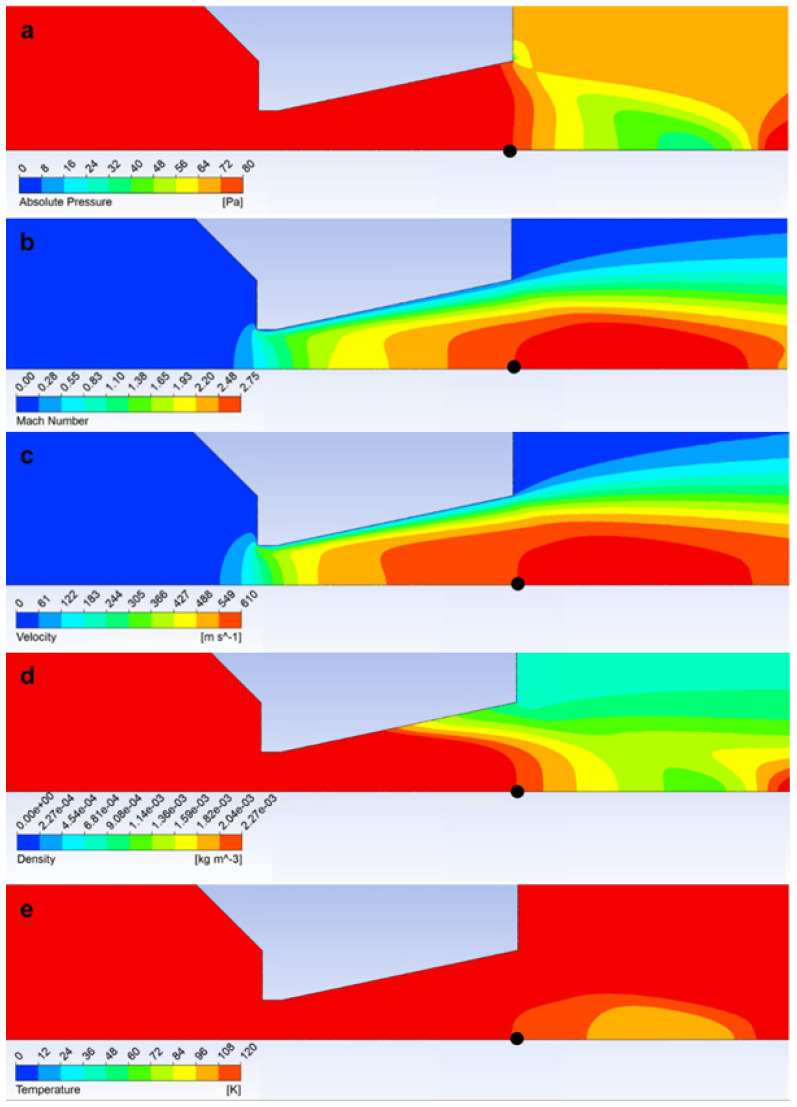
Layout of static pressure (**a**), Mach number (**b**), velocity (**c**), density (**d**), and temperature (**e**).

Figure 7 shows the actual quantities obtained by mathematical–physics analysis using the Ansys Fluent system static pressure (Figure 7a), Mach number (Figure 7b), velocity (Figure 7c), density (Figure 7d), and temperature (Figure 7e) at the end of the nozzle (black point in Figure 7a–e) corresponding to the theoretical values according to Table 1.

The entire work of the research team uses a modern method of combination:

- Experimental measurement using sensors;
- Mathematical–physics analyses;
- Physical theory.

In the next step, further mathematical–physics analyses were performed on the tuned mathematical–physics model in the Ansys Fluent system. This mathematical–physics model was tuned using experimental measurements and the theory of isentropic one-dimensional flow physics (Equations (1)–(6)).

These additional mathematical–physics analyses were performed on the nozzle with the same dimensions as in the previous case. The only difference was that the angle X was changed (Figure 6). It is a mapping of the critical supersonic flow in the nozzle and above the nozzle on the axis of the primary electron beam because the calculation state may not be the most advantageous for the effective passage of the primary beam concerning the loss of this beam through iterations of electrons with gas molecules.

The variants shown in Table 2 were chosen for the analyses presented in this article.

For all variants, the boundary conditions were set according to Figure 5.

### 5.2. Evaluation of Nozzle Opening Variants

For analyses of the effect of changing the size of the nozzle angle, the tuned Ansys Fluent system was used. It was placed behind the aperture, and experiments on the effect of supersonic flow in the nozzle and behind the nozzle were carried out.

**Figure 8:**
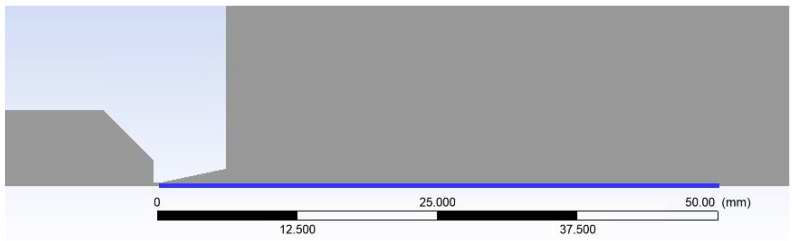
Axis of the flow (blue line—Path).

In the first step of the evaluation, the basic quantities on the flow axis were plotted (Figure 8—blue line). The flow axis is called Path in the following graphics.

**Figure 9:**
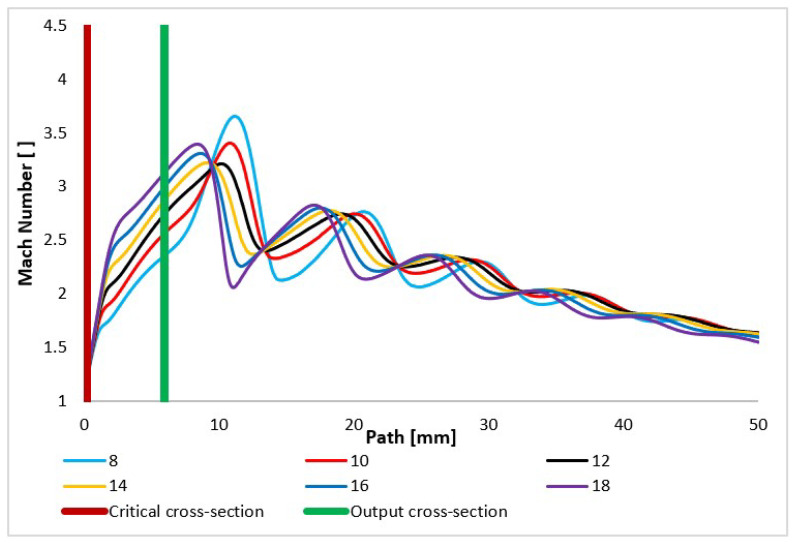
Mach number layout of each variant of the nozzle in the primary electron beam path.

The Mach number and the related velocity quantities prove that the calculated cross-section with an angle of 12° copies the uniform gas expansion beyond the nozzle. This calculated cross-section is marked red in Table 3. The calculated state is compared with over-expanded and under-expanded variants of nozzle angles. Similarly, in the nozzles with a larger opening, the velocity is uniform through the nozzle throat, despite allowing for over-expansion and gaining higher speed in the nozzle and earlier termination of expansion behind the nozzle. On the contrary, the nozzle variants with a smaller angle are under-expanded in the nozzle. There is an additional gas expansion behind the nozzle, which is especially noticeable for the nozzle with the smallest angle of 8° due to the significantly increased speed behind the nozzle. This fact significantly affects the course of static pressure, which is of key importance for the scattering of the primary electron beam and thus for the resulting sharpness of the obtained image in the ESEM, as discussed below. The course of the Mach number significantly affects the course of other monitored variables (Figure 9).

**Figure A1:**
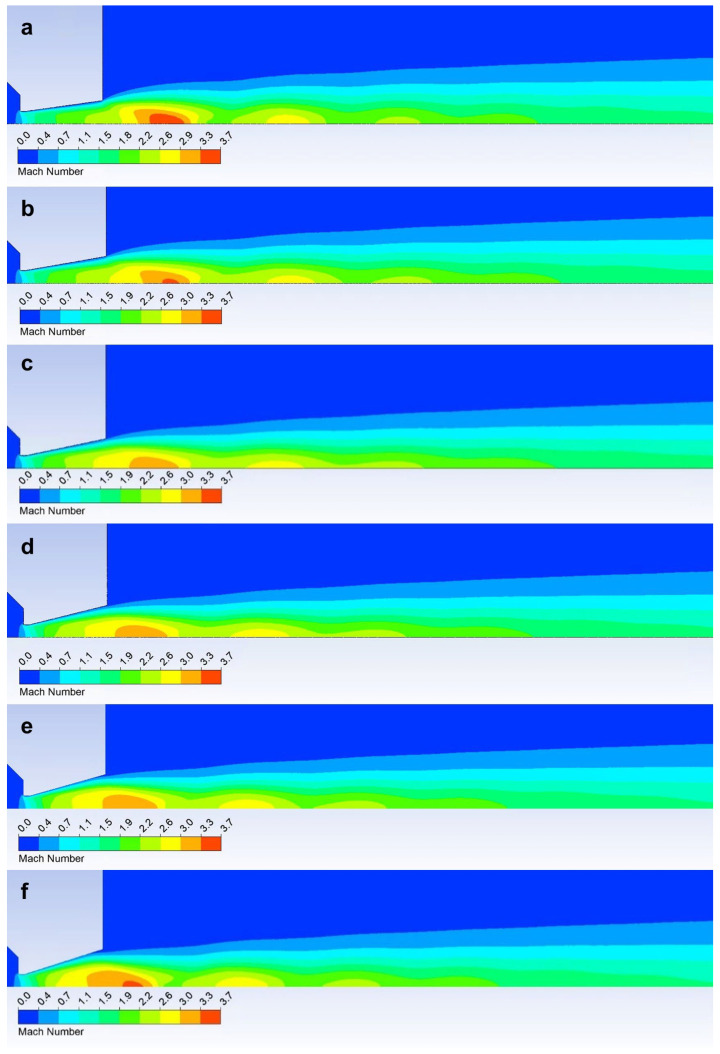
Graphical distribution of Mach number in the nozzle opening of 8° (**a**), 10° (**b**), 12° (**c**), 14° (**d**), 16° (**e**), and 18° (**f**).

A graphical distribution of Mach numbers is in Appendix A (Figure A1).

**Figure 10:**
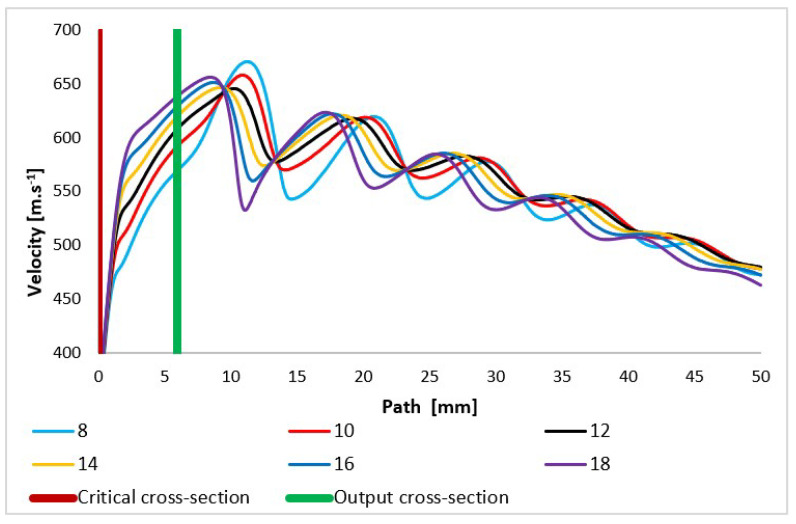
Velocity layout of each variant of the nozzle in the primary electron beam path.

The flow velocity course is very similar to the Mach number course (Figure 10) but is still influenced by the fact that in the critical flow area, the conditions for sound propagation in the given environment vary greatly. It is very dependent on temperature and pressure, as discussed below. These quantities change very significantly in a supersonic flow area with high gradients.

For the speed of sound in an ideal gas, Equation (13) applies.

(13) $$c=\sqrt{\kappa \frac{p_{0}}{\rho_{0}}}\left(1+\frac{1}{2}\gamma t\right)$$

where *p*_0_ is gas pressure at 0 °C, *ρ*_0_ is the relevant density, *κ* is the gas constant, *t* is the temperature, and *γ* is the thermal expansion for the gas.

The velocity of the body can be further calculated from the equation for calculating Mach number (Equation (14)).

(14) $$M=\frac{v}{c}$$

where *v* is the speed of motion of the body; *c* is the speed of sound.

**Figure 11:**
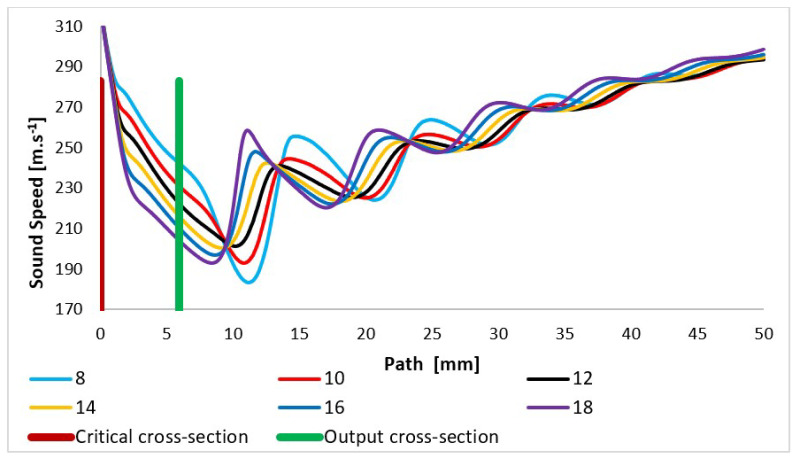
Speed of sound layout of each variant of the nozzle in the primary electron beam path.

Due to the pressure and temperature in the expansion area changing sharply, the quantity of the speed of sound propagation in the given environment changes on a given path, especially in the nozzle area and in the area of additional expansion behind the nozzle, as already mentioned (Figure 11).

In practice, the static temperature values are scanned on the path (Figure 8) during experimental measurements using temperature sensors (custom-made K-type thermocouple with a diameter of 3 mm), where, in the supersonic region with a perpendicular shock wave, the static temperature is obtained from the stagnation temperature using Equation (15) because the temperature sensor senses stagnation temperature at speeds higher than 50 m·s^−1^ [41]:

(15) $$\frac{T}{T_{0}}=\left(1+\frac{\gamma -1}{2}\right)M^{2}$$

where *T* is static temperature, *T*_0_ is total temperature, *γ* is Poisson constant, and *M* is Mach number.

**Figure 12:**
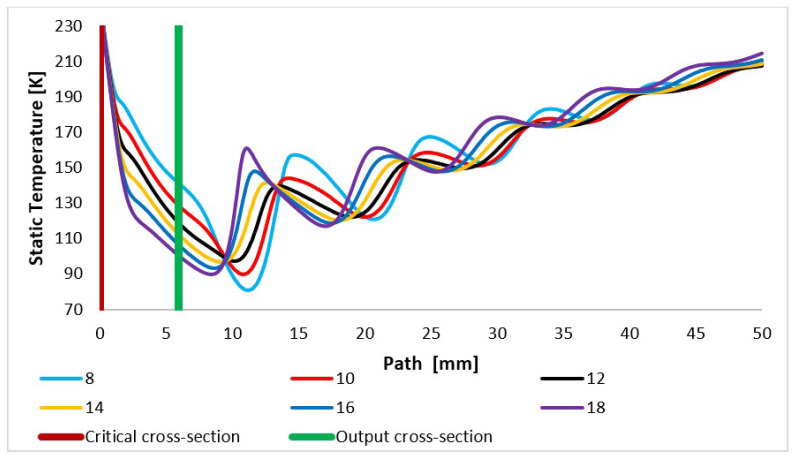
Static temperature layout of each variant of the nozzle in the primary electron beam path.

Due to the interconnection with the flow velocity and static pressure, the static temperature pattern depicts the character of the flow according to the under-expansion or over-expansion of the gas flow. In the case of an over-expanded nozzle, the gas temperature drops sharply from the critical point to the lowest values, and at the end of the expansion, shortly after the nozzle throat, there is a sharp increase. On the other hand, the under-expanded nozzle in the nozzle area shows a more gradual decrease in temperature, while in the area of additional expansion, there is another sharp drop in temperature with the highest minimum of all variants, and the temperature rises again at a greater distance—up to 4 mm further than the overexpanded 18° variant (Figure 12).

**Figure A2:**
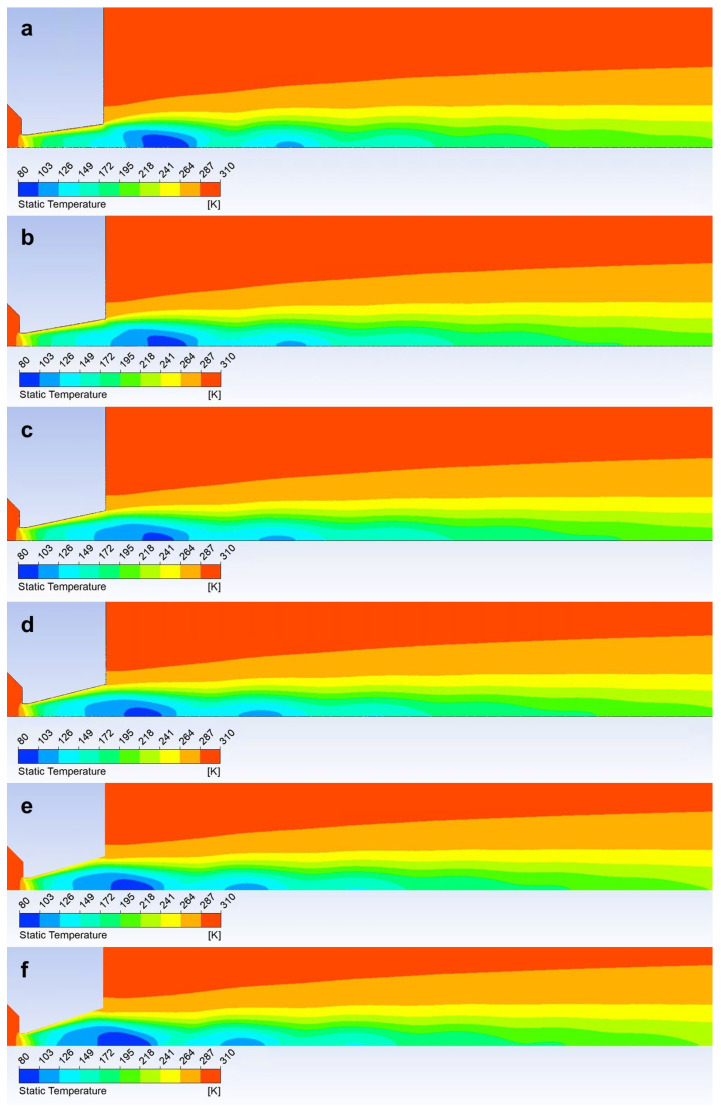
Graphical distribution of static temperature in the nozzle opening of 8° (**a**), 10° (**b**), 12° (**c**), 14° (**d**), 16° (**e**), and 18° (**f**).

A graphical distribution of static temperature is in Appendix A (Figure A2).

**Figure 13:**
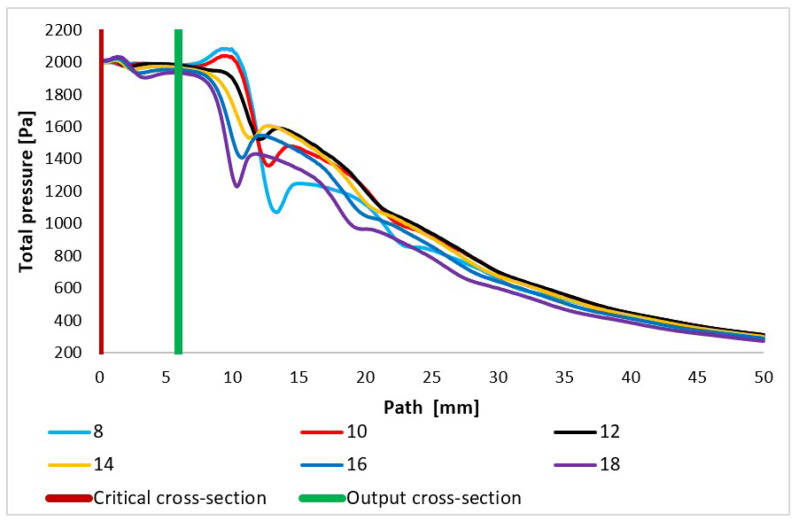
Total pressure layout of each variant of the nozzle in the primary electron beam path.

In addition, the total pressure and static pressure values are scanned in the path (Figure 8) during experimental measurements using pressure sensors (multirange differential pressure sensors for gases and air DPS 300), as these two quantities allow for the flow velocity to be evaluated based on the Pitot tube principle [42,43,44,45]. The assumed total pressure is shown in Figure 13 and again shows a completely different course, especially behind the nozzle in the variants of under-expanded and over-expanded nozzles [46,47]. As is discussed below, these facts are of key importance to the function of the electron microscope.

**Figure 14:**
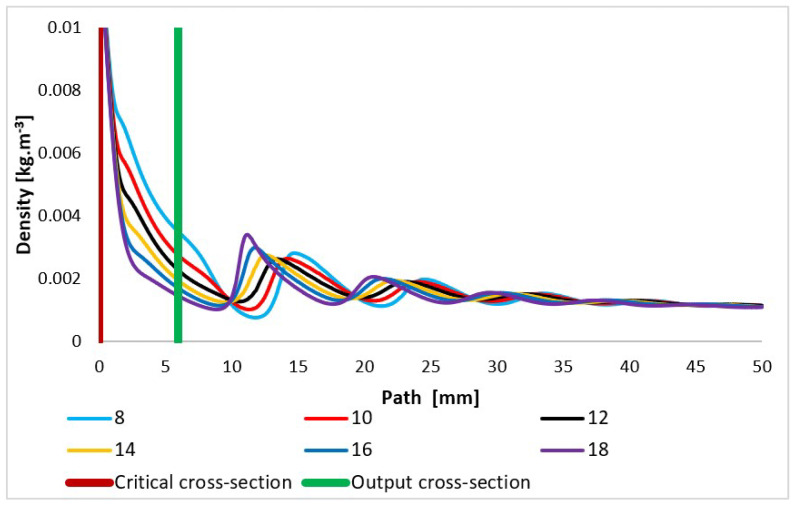
Density layout of each variant of the nozzle in the primary electron beam path.

**Figure 15:**
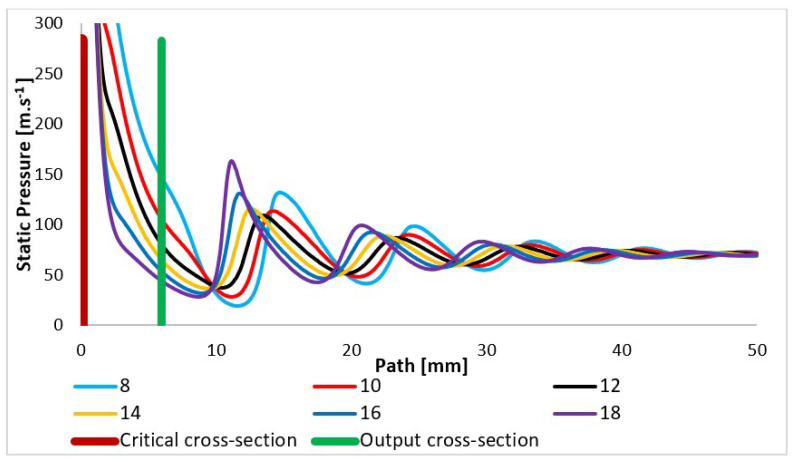
Static pressure layout of each variant of the nozzle in the primary electron beam path.

The courses of the quantities density (Figure 14) and static pressure (Figure 15) have the same character and are very closely related to the course of the Mach number. With an increase in the velocity of gas flow, a sharp decrease in values is evident in density and static pressure. In compact nozzles with angles of 8° and 10°, due to under-expanded flow with a slower increase in velocity, a slower decrease in static pressure and density in the nozzle area is noticeable. Due to additional expansion behind the nozzle, there is a further pressure drop in these variants and the Mach disk is visible at a distance of about 15 mm, while in overexpanded nozzles with a larger opening, there is a much steeper pressure drop in the nozzle and already a noticeable sharp increase in pressure and density at a distance of 12 mm in the Mach disk. For the nozzle with the largest opening, the increase in static pressure and density is significantly the highest of all variants.

**Figure A3:**
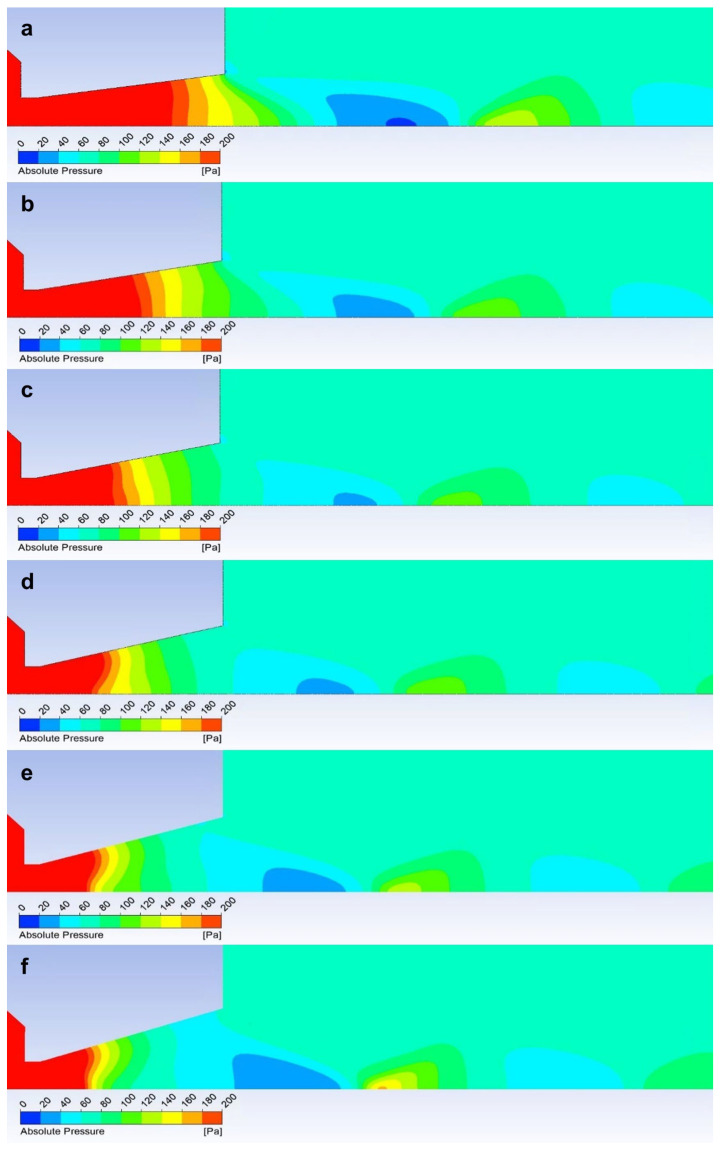
Graphical distribution of static (absolute) pressure in the nozzle opening of 8° (**a**), 10° (**b**), 12° (**c**), 14° (**d**), 16° (**e**), and 18° (**f**).

**Figure A4:**
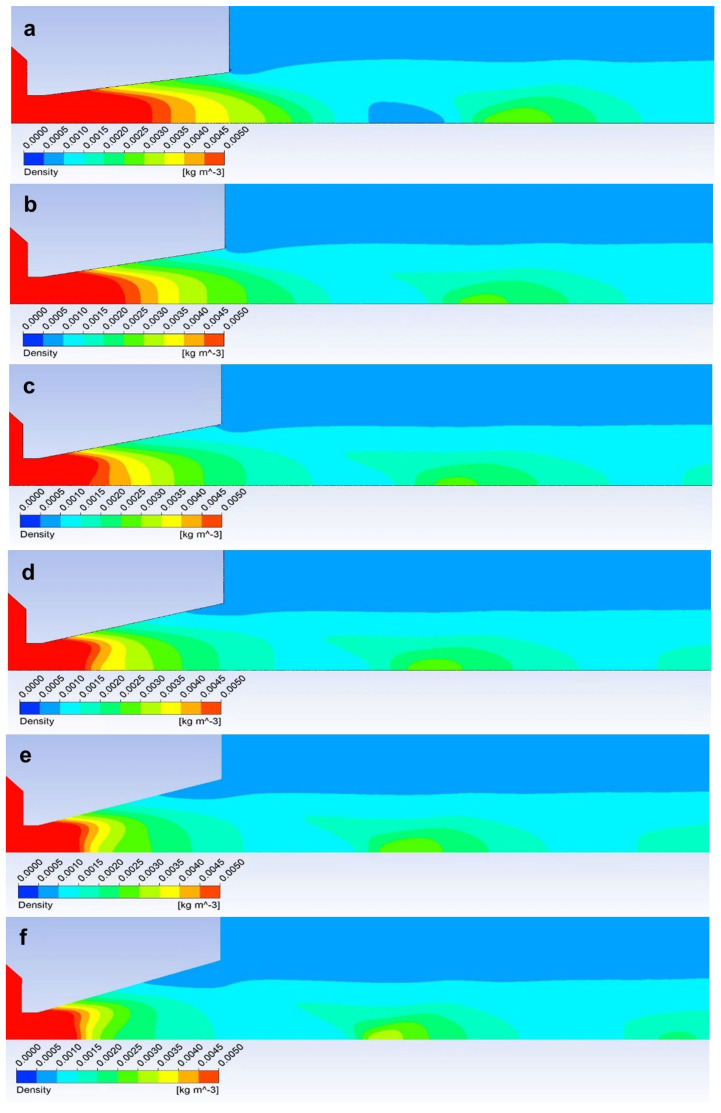
Graphical distribution of density in the nozzle opening of 8° (**a**), 10° (**b**), 12° (**c**), 14° (**d**), 16° (**e**), and 18° (**f**).

A graphical distribution of static pressure and density is in Appendix A (Figure A3 and Figure A4).

These facts are of fundamental importance in determining the most optimal variant of favorable static pressure conditions for the passage of the primary electron beam. From the course of the curves, it is already clear that a sharp drop in static pressure and density per aperture in the 18° open nozzle is decisive and advantageous to the ESEM’s functioning. This decrease is replaced by a subsequent larger increase at the end of the supersonic flow and an increase in pressure in the Mach disk compared to other variants.

### 5.3. Evaluation of Electron Dispersion for Each Variant

In an electron microscope, the interaction of the electron beam and the gas scatters the primary electrons. In electron microscopes operating at higher pressures in the specimen chamber (ESEM), a certain part of the electron remains in the original trace even after passing through the gaseous medium. This part of the electron then interacts with the sample to create a signal similar to a conventional electron microscope.

As the primary electron beam passes through a medium with higher pressures of gases, the electrons interact with atoms and molecules of gases. Electrons in these collisions can lose some of their energy and change the direction of their path. If the average number of collisions *M* in a gaseous medium is small, the resulting deviation of the electron from the original beam path in the plane of the preparation *r* is also small. We can then lay the length of the electron’s path equal to the thickness of the gas layer *d* through which the electron is moving. The average number of collisions per electron can be determined from Equation (16) [48]:

(16) $$M=\frac{\sigma_{T}Pd}{kT}$$

where *σ_T_* is the total gas gripping cross-section, *P* is static pressure, *d* is the thickness of the gas layer through which the electron passes, *k* is the Boltzmann constant, and *T* is the absolute temperature.

Then, the following applies for the value of *M*:

- *M* < 0.05: there is minimum beam dispersion of up to 5%;
- *M* = 0.05–3: there is partial dispersion in the range of 5–95%;
- *M* > 3: there is complete dispersion above 95%.

Gripping cross-section *σ_T_* is defined as the close area of a gas particle. A collision occurs if the electron is at this point during its passage. Thus, the gas gripping cross-section does not depend only on the type of gas but also on the accelerating voltage. For our case, nitrogen, together with an accelerating voltage of 10 keV, was chosen. In this case, according to [49], the gripping cross-section *σ_T_* = 2 × 10^−21^ [m^2^].

**Figure 16:**
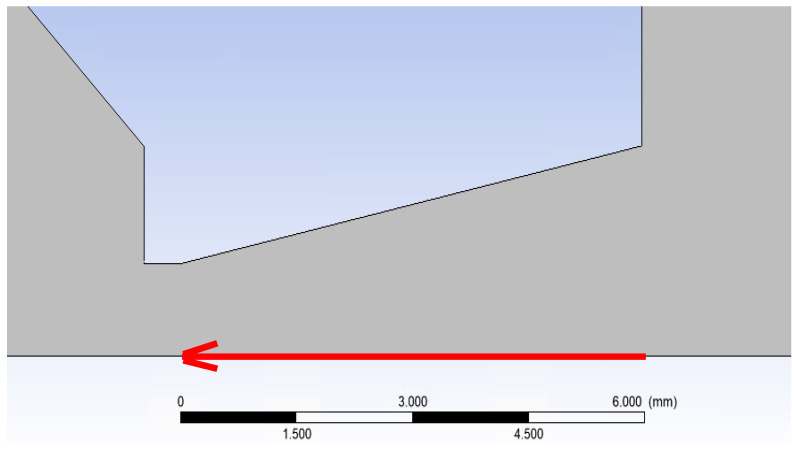
Direction of primary electron beam path.

The path on which the primary electron beam passes through the nozzle in a real ESEM is shown in Figure 8. The nozzle is 5.941 mm long. The primary electron beam runs against the flow direction, as indicated by the arrow in Figure 16.

**Figure 17:**
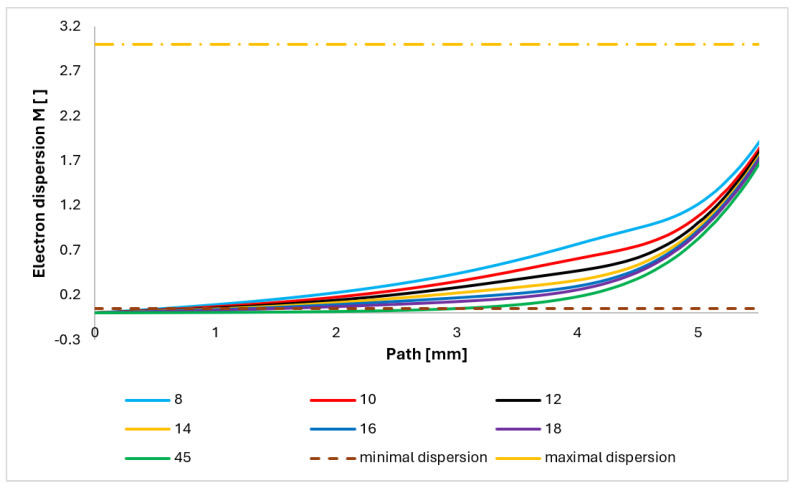
Course of electron dispersion value on the primary electron beam path.

Figure 17 shows the course of the electron scattering value M on this path. It is noticeable that above the value of *M* = 0.05, the values of the lower angle tend to rise faster. It is, therefore, preferable to choose a larger angle, as the magnitude of the M scatter has a direct effect on the resulting sharpness of the image. The key is to ensure that the course of the primary beam takes place at the lowest possible M level in the construction of differentially pumped chambers.

A simplified analysis to compare the ratio of beam dispersion size for each variant can be performed using the probability of electron scattering, which is derived from a given pressure occurring on the path of the primary electron beam. The electron is directly proportional to the product of the pressure values and the distance the electron travels. The significant initial dependence of pressure on distance on the path (Figure 8) can be expressed using the integral (Equation (17)).

(17) $$Pd=\int p\left(x\right)dx$$

**Figure 18:**
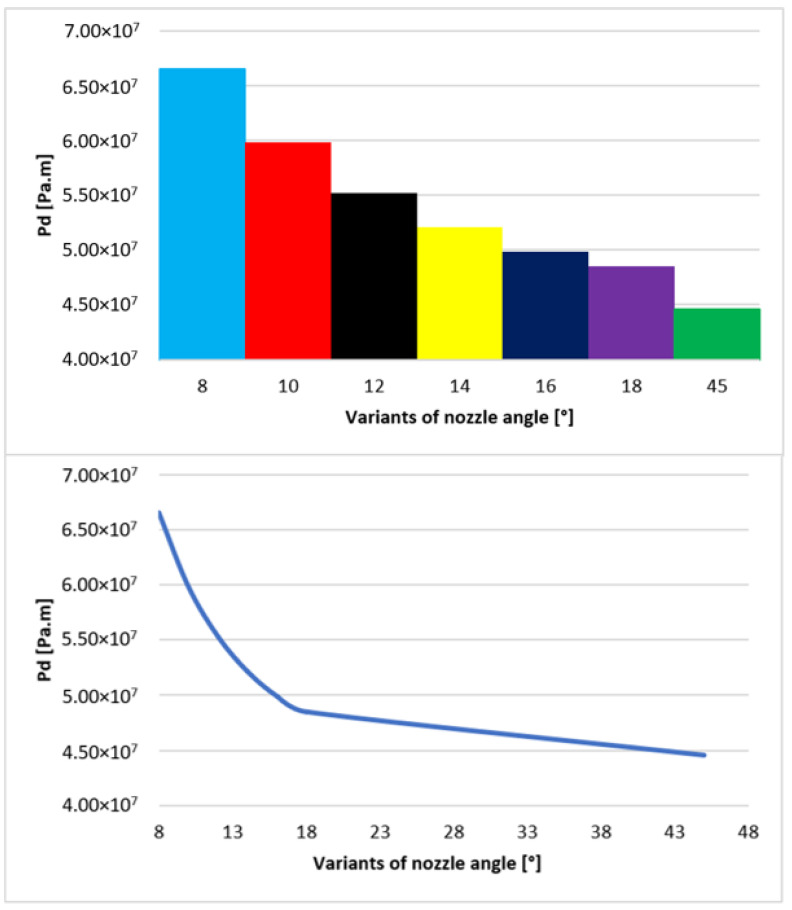
Graphic of probability of electron dispersion in each variant.

From the displayed values of electron dispersion of each nozzle opening (variants of nozzle angle (Figure 6)), it is clear that with increasing opening of the nozzle, the probability of electron dispersion decreases (Figure 18). This has a direct effect on the number of incident electrons on the element being tested and the overall image transfer. Therefore, it is desirable to keep the dispersion as low as possible.

The results presented in Figure 18 clearly show that the shape of the over-expanded open nozzle is preferable for the course of the primary electron beam. Figure 18 further shows that the ratio between the nozzle opening and the dispersion tendency is not linear but exponential. A variant of nozzle opening with a significantly higher value of 45° was added. This variant helped to depict the character of the curve (Figure 18), from which it is already possible to approximate values between 20° and 45°, as well as values above 45°. It is evident that from about 20°, the effect of the opening is not as marked as in values up to 20°.

**Figure 19:**
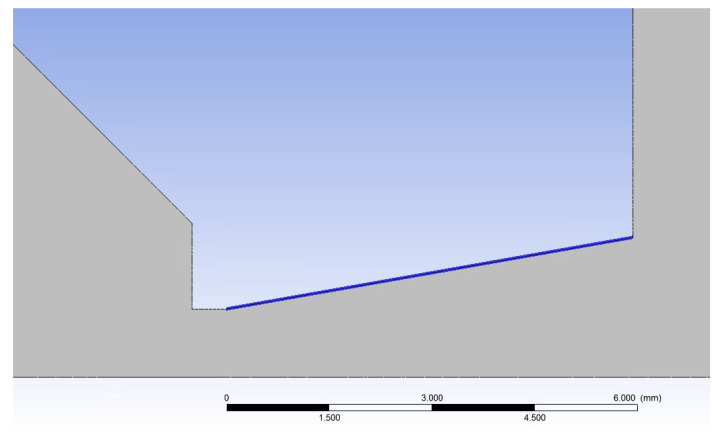
Examined nozzle surface (blue line—Path).

The temperature on the nozzle surface (Figure 19—blue line; further called Path in the graphics) was also scanned using temperature sensors (custom-made K-type thermocouple with a diameter of 3 mm) during experimental measurements. So, this helped to evaluate the flow pattern of the under-expanded and over-expanded nozzle.

**Figure 20:**
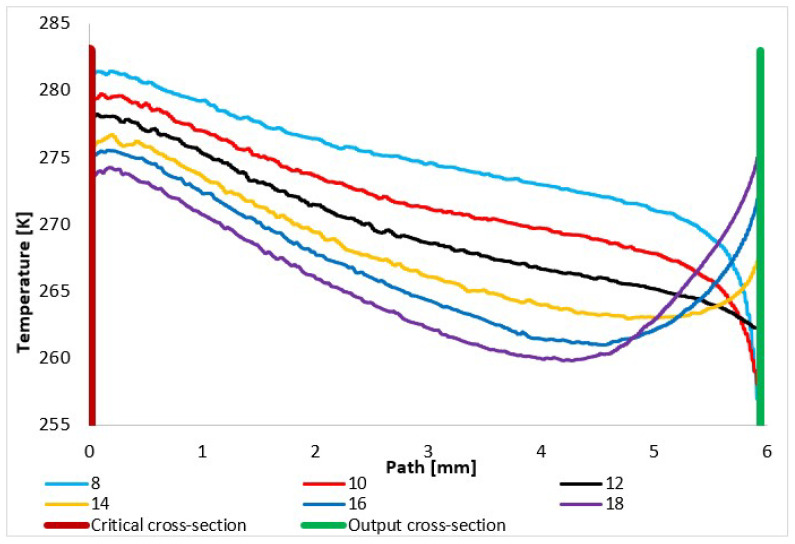
Temperature layout of each variant of the nozzle in the primary electron beam path.

Mathematical–physics analyses using the Ansys Fluent system show the assumption of a different temperature curve on the nozzle surface (Figure 20). This occurs because of the very different flow velocities in all variants due to the different expansions of the gas behind the aperture. Line fluctuations are caused by slight turbulence when braking the gas on the nozzle wall in slip flow mode due to low pressure.

**Figure 21:**
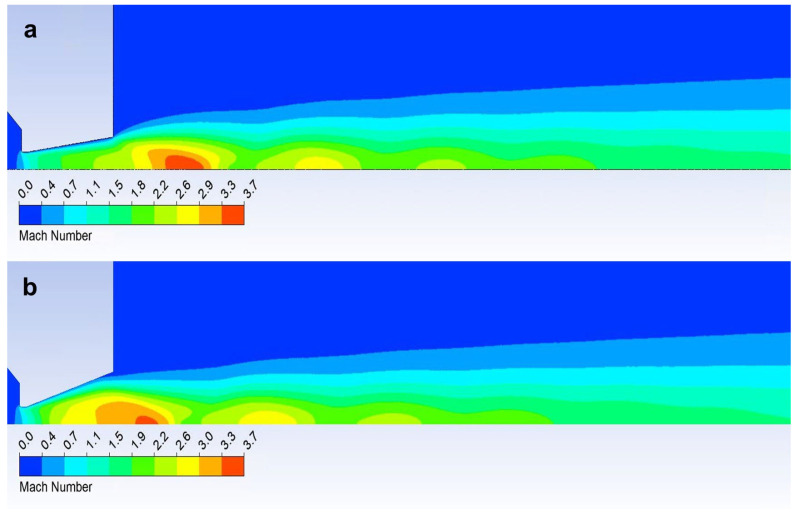
Mach number layout of over-expanded nozzle 8° (**a**) and under-expanded nozzle 18° (**b**).

The temperature course proves that the nozzle with a calculated cross-section of 12° has a uniform temperature curve on the wall, demonstrating a decreasing temperature corresponding to the increase in velocity. Conversely, the under-expanded 8° nozzle has the highest temperature course with a sharp drop at the end of the nozzle, where there is a further increase in velocity, and thus a decrease in temperature (Figure 20) [50,51]. This is visible in Figure 21a, where there is a distinct area of supersonic flow behind the nozzle. The velocity course shows a sharp increase and the highest Mach number values towards the end of the nozzle, behind the nozzle, and vice versa—lower velocities—in the first half of the nozzle. Figure 21a also shows that behind the nozzle, the velocity flow of gas expands in width before the first braking of the velocity occurs and increases again. This pulsation, as seen in Figure 10, causes oblique shock waves.

On the other hand, an over-expanded nozzle demonstrates a course with the lowest temperature with a temperature increase at the end of the nozzle. Here, the velocity flow is torn from the nozzle wall [52,53]. This is evident in Figure 21b, where the Mach number distribution is shown. Compared to the previous case, on the contrary, it can be seen that the striking area of the Mach number is inserted into the nozzle. The sharp increase in velocity compared to the previous case occurs just behind the aperture. Therefore, the temperature course on the wall is lower (Figure 20). However, due to the rapid increase in velocity behind the aperture in this variant, the velocity flow is compressed at the nozzle throat. Figure 21b shows that the flow velocity at the nozzle throat is very small compared to the main flow. The main flow is torn away from the wall. Here, it is heading towards the first braking and subsequent pulsation caused by oblique shock waves. For this reason, we can detect a temperature increase on the nozzle wall using sensors in the experimental chamber, because the velocity of the flowing gas is low due to the compression of the velocity flow.

**Figure 22:**
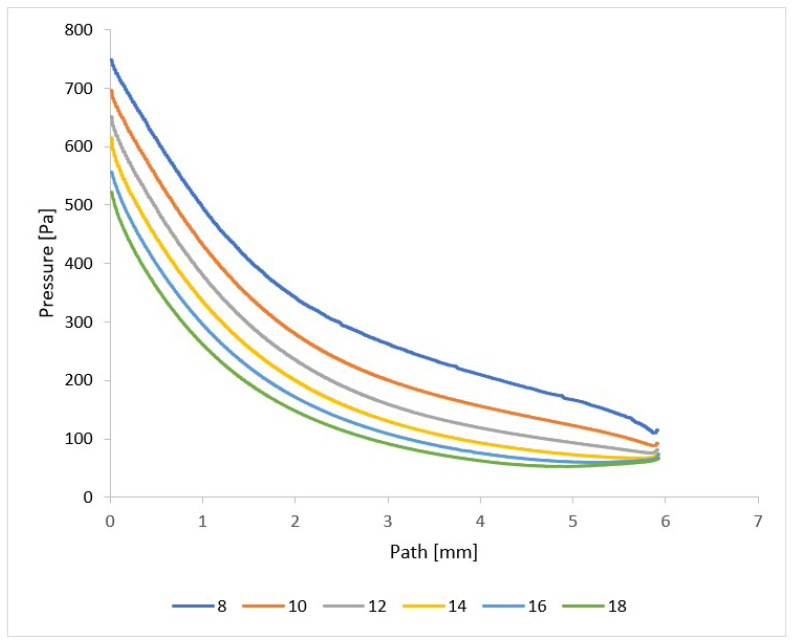
Pressure layout of each variant of the nozzle in the primary electron beam path.

The presented results are suitably complemented by a pressure distribution analysis of the nozzle wall. The previous description of the temperature distribution on the nozzle and the course of velocity given by the Mach number is directly related to the pressure distribution. In the experimental chamber (Figure 2), the pressure on the nozzle walls are monitored by temperature sensors (custom-made K-type thermocouple with a diameter of 3 mm). The expected pressure distribution is shown in Figure 22 for each variant. Compared to the temperature distribution shown in Figure 20 for pressure, there are no such significant temperature changes at the end of the nozzle opening, and up to a length of about 6 mm for each variant, there is a uniform pressure drop. After that, a significant difference can be seen. In the case of nozzles with a small opening, the pressure decreases further, but in the case of nozzles with a large opening, such as the opening angles of 16° and 18°, there is also a slight increase. Again, the reason is the separation of the gas flow from the nozzle wall at the end of the nozzle due to the greater backpressure of the surrounding gas in the case of an overexpanded nozzle shape. In the experiment, the values taken from the nozzle walls confirm for all variants of nozzle angles whether under-expanded, calculated, or over-expanded in shape. Using verification for mathematical–physics analyses, they serve as a basis and starting point for further research for suitable nozzle shaping for differentially pumped ESEM chambers.

## 6. Conclusions

This paper analyzed the issue of aperture with the nozzle in ESEM vacuum chambers, i.e., the specimen chamber and differentially pumped chamber, where the aperture causes a large pressure gradient between individual chambers. These large pressure gradients cause critical gas flows containing supersonic flows with large pressure gradients. Next, a combination of experimental measurements in conjunction with the theory of one-dimensional isentropic flow and mathematical–physics analyses were presented as a tool for the development of a nozzle ensuring optimal pressure distribution in the axis of the primary electron beam that passes through the environment to minimize electron collisions between the beam and air molecules. Furthermore, the effect of the change in nozzle angle size on the character of the supersonic flow and its effect on the resulting pressure in the path of the primary beam was analyzed. The results also serve as a basis for the upcoming experiment. The flow pattern of the nozzle was analyzed on a tuned mathematical–physics model, with dimensions determined using the theory of one-dimensional isentropic flow for a computational cross-section with an angle of 12°. Then, this calculated state of the nozzle was compared to the flow pattern of under-expanded nozzles with aperture angles of 8° and 10° and over-expanded nozzle variants with opening angles of 14°, 16°, and 18°. Finally, the results clearly show that the shape of the over-expanded open nozzle is preferable for the course of the primary electron beam, where the character of the flow with a rapid and very high increase in velocity in the nozzle immediately after the aperture creates a large pressure drop. Although the re-increase in pressure occurs earlier than in under-expanded nozzles, more open nozzles are favorable for an overall environment that provides conditions for the lowest possible electron beam dispersion.

## Figures and Tables

**Table 1:** Comparison of results obtained from the theory of isentropic one-dimensional flow (Equations (1)–(6)) with results from the mathematical–physics analysis in Ansys Fluent system.

	Theory of One-Dimensional Isentropic Flow	ANSYS Fluent
P_v_/P_0_ [–]	0.04	0.04
M_v_ [–]	2.75	2.74
T_0_ [K]	297.15	297.15
V_v_ [m·s^−1^]	600	608
ρ [kg·m^−3^]	0.00234	0.00227
T_v_ [K]	118.27	118
P_v_/P_0_ [–]	0.04	0.04

**Table 2:** Angle variants of the nozzle.

Angle X of Nozzle Opening from Axis [°]	Radius of Output Cross-Section [mm]	
8	1.8	Expected additional expansion
10	2	Expected additional expansion
12	2.2	Computational cross-section
14	2.5	Expected additional compression
16	2.7	Expected additional compression
18	2.9	Expected additional compression

**Table 3:** State quantities of individual variants of nozzle opening.

Angle X of Nozzle Opening from Axis [°]	*M_v_* [–]	*V_v_* [m·s^−1^]	*ϱ_v_* [°]	*Tv* [K]
8	2.35	570	0.0035	141
10	2.56	592	0.0028	128
12	2.74	608	0.0023	118
14	2.87	620	0.0019	112
16	3	629	0.0017	106
18	3.13	639	0.0015	100

## Data Availability

The data presented in this study are available on request from the corresponding author.
